# Supplementary figures and images for: Monitoring of Staphylococcus epidermidis biofilm formation on platelet storage bag surfaces
Source: PLoS One. 2025 Oct 22;20(10):e0333558. doi: 10.1371/journal.pone.0333558 (PMC12543116; doi:10.1371/journal.pone.0333558)

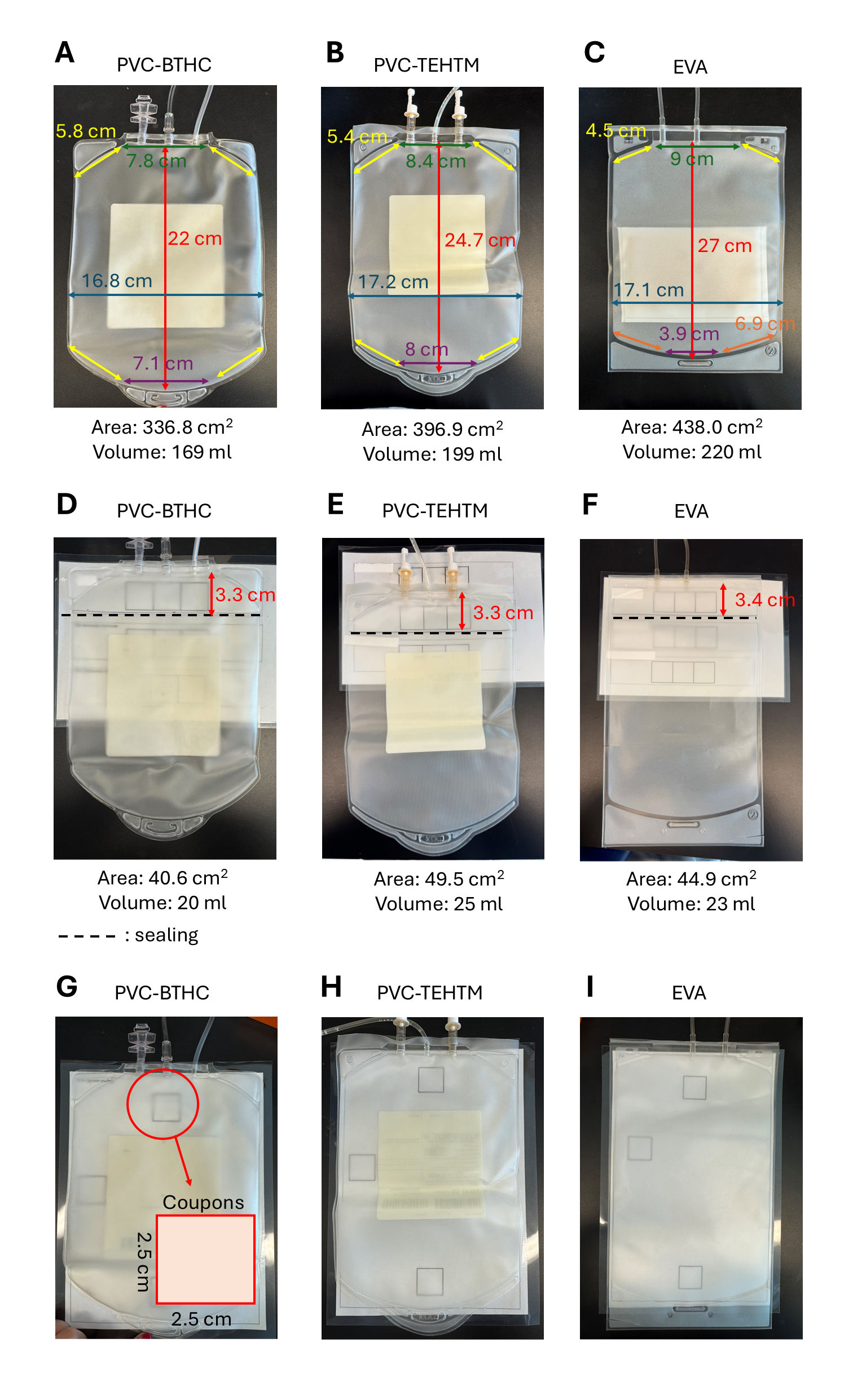

Supplement: S1 Fig — Two formats are shown: full-size bags, used for experiments involving TSB and plasma, and reduced-volume bags, created by heat-sealing full-size bags, used for experiments with PCs. This figure illustrates the physical dimensions of the PVC-BTHC (A, D), PVC-TEHTM (B, E) and EVA (C, F) bags (cm), the internal surface areas available for gas exchange (cm² for surface areas), and the total volume (ml) after the addition of the tested storage medium. Specific regions where 2.5 × 2.5 cm coupons were cut for CV experiments are shown on the reduced-volume PC bags (D, E, F) and the full-size PC bags (G, H, I). (TIF) [file pone.0333558.s001.tif]

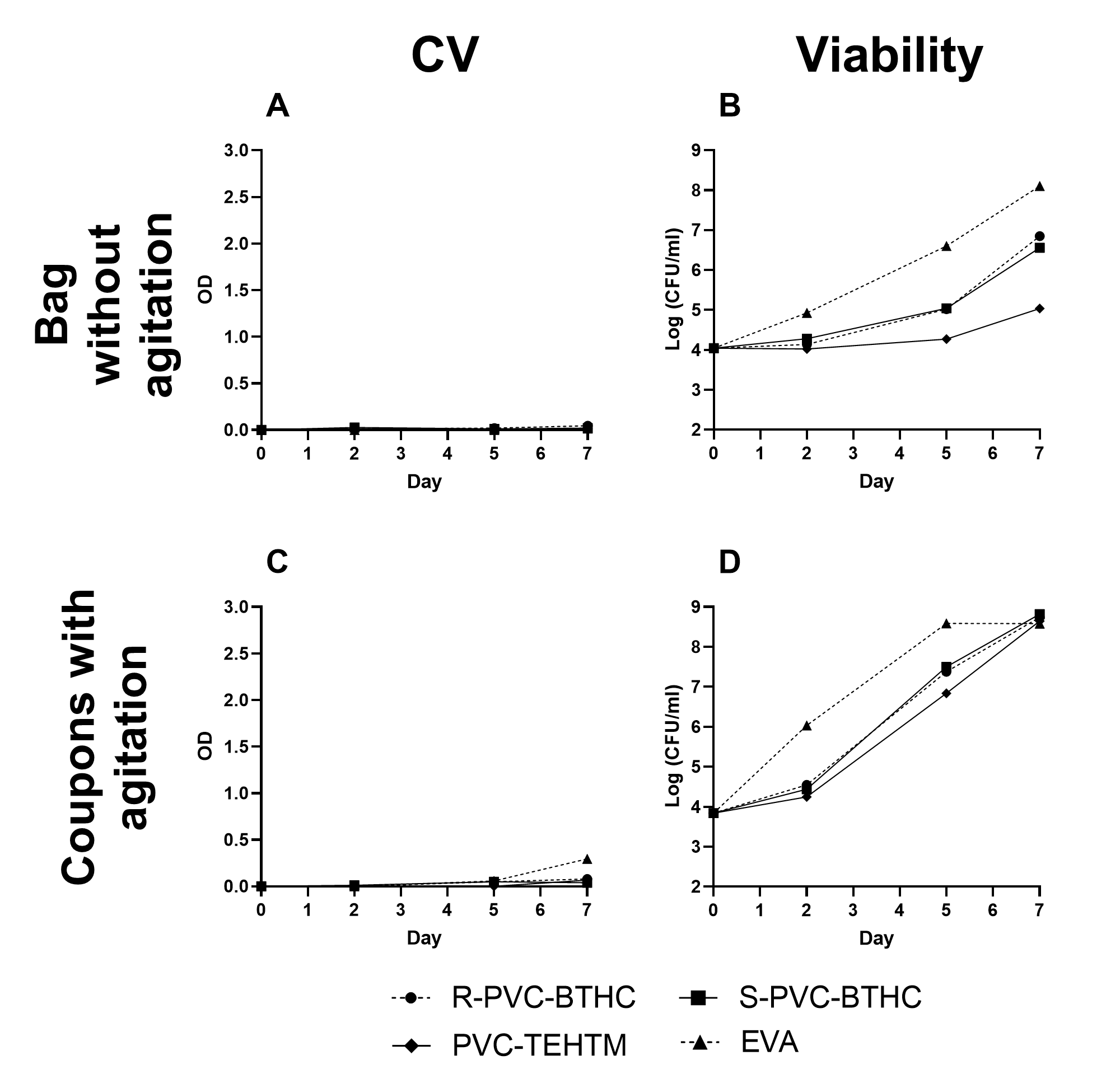

Supplement: S2 Fig — Mean OD by CV assay (A, C) and mean log bacterial concentration (log CFU/ml) (B, D) are shown for day 2, 5, and 7 after incubation in bags without agitation (A, B) and on coupons with agitation (C, D) in TSB (n = 1). R-PVC-BTHC (), S-PVC-BTHC (), PVC-TEHTM () and EVA () are represented. (TIF) [file pone.0333558.s003.tif]
