# Supplementary material for: Monitoring of Staphylococcus epidermidis biofilm formation on platelet storage bag surfaces
Source: PLoS One. 2025 Oct 22;20(10):e0333558. doi: 10.1371/journal.pone.0333558 (PMC12543116; doi:10.1371/journal.pone.0333558)
Supplement: S1 File — Biofilm quantification (OD) and bacterial counts from S. epidermidis assays under different incubation conditions: agitation, reduced oxygen, and static incubation at varying concentrations (see Table 1 and S2 Fig). (DOCX) [file pone.0333558.s002.docx]

**Monitoring of *Staphylococcus epidermidis* biofilm formation on platelet storage bags surfaces**

Jolianne Matte^1,2^, Sahra Fonseca^1^, Jonathan Robidoux^1^, Steve J. Charette^2,3^, Marie-Pierre Cayer^1*^ and Danny Brouard^1,2*^

**Supporting information**

Additional tests were conducted in TSB to determine whether agitation and oxygen availability were the key factors contributing to preventing biofilm formation in PC bags. *S. epidermidis* was inoculated on coupons and in PC bags using the same methods as previously described (sections Bacterial strains and growth conditions, Inoculation of bacteria on coupons and Inoculation of bacteria in PC storage bags), but with different incubation conditions. Inoculated coupons of all PC bag materials were incubated under agitation on the same platelet agitator used for the bags (**S2A and S2C Figs**). Additionally, a test was carried out incubating R-PVC-BTHC coupons in reduced oxygen levels, using GasPak EZ anaerobe pouch system (BD, cat. 260683) (**Table 1**). Finally, inoculated PC bag assays were incubated without agitation at a bacterial concentration of 10^4^ CFU/ml (**S2B and S2D Figs**). An additional assay was conducted with R-PVC-BTHC bags at a bacterial concentration of 10^7^ CFU/ml (**Table 1**).

| **Table 1.** **Biofilm formation and bacterial concentration on R-PVC-BTHC with reduced oxygen levels or high initial concentration** | | | | |
| --- | --- | --- | --- | --- |
| **Incubation conditions** | **OD** | | **Bacterial concentration**  **(log CFU/ml)** | |
|  | **Day 0** | **Day 7** | **Day 0** | **Day 7** |
| **Reduced oxygen levels**  (coupons) | 0.0 | 0.02 | 3.9 | 6.5 |
| **Static**  (PC bag) | 0.0 | 2.8 | 7.0 | 7.5 |
|  | | | | |
