# Supplementary material for: Monitoring of Staphylococcus epidermidis biofilm formation on platelet storage bag surfaces
Source: PLoS One. 2025 Oct 22;20(10):e0333558. doi: 10.1371/journal.pone.0333558 (PMC12543116; doi:10.1371/journal.pone.0333558)
Supplement: Attachment — Submitted filename: Response to Reviewers.docx [file pone.0333558.s005.docx]

PONE-D-25-18826

Monitoring of Staphylococcus epidermidis biofilm formation on platelet storage bag surfaces

*Dear Dr. Hwang,*

*Thank you very much for your thoughtful feedback and for the opportunity to revise our manuscript submitted to PLOS ONE. We appreciate the time and effort you and the reviewers have dedicated to evaluating our work.*

*We have addressed all the comments and suggestions provided, and we have ensured that the revised manuscript meets the journal’s formatting and submission requirements.*

*Please do not hesitate to let us know if there are any additional points, we should consider during the revision process.*

*Thank you again for your guidance and support.*

**Responses to Editor's Comments**

**1. Please ensure that your manuscript meets PLOS ONE's style requirements, including those for file naming.**

**Editor's request completed:** Renamed the figure files to comply with the journal's file naming conventions. Adjusted the font size of the figure titles within the manuscript to align with the required formatting guidelines.

**2. PLOS requires an ORCID iD for the corresponding author in Editorial Manager on papers submitted after December 6th, 2016.**

**Editor's request completed**: As requested, the corresponding author has registered for an ORCID iD and successfully validated it in Editorial Manager.

**3. We notice that your supplementary figures are uploaded with the file type 'Figure'.**

**Editor's request completed:** The legend for Figure S1 and S2 in the supporting information has been moved to the reference list. Figure S1 and S2 has been uploaded to the “Supporting Information” category.

**4. Please remove all personal information, ensure that the data shared are in accordance with participant consent, and re-upload a fully anonymized data set.**

**Editor's request completed:** We would like to clarify that no raw or individual-level data files were shared or uploaded as part of our submission. Therefore, there are no personal data or spreadsheet columns to anonymize or remove. All data presented in the manuscript are aggregated and fully anonymized in accordance with participant consent and ethical guidelines.

Additionally, we have ensured that all personal information related to the authors has been removed from the title page of the manuscript, in accordance with the journal’s anonymization requirements.

Please let us know if further clarification is needed.

**5. Please include captions for your Supporting Information files at the end of your manuscript, and update any in-text citations to match accordingly.**

**Editor's request completed:** The legend for Figure S1, now Figure S2, has been moved to the end of the manuscript.

**Responses to Reviewers' Comments**

We would like to sincerely thank reviewers for their careful review of our manuscript and for the constructive comments and suggestions they provided. Your feedback has been extremely valuable in helping us improve the quality and clarity of our work.

We have carefully addressed each of your points in the revised version of the manuscript, and we provide detailed responses below to explain the changes made.

We greatly appreciate your time and effort in reviewing our submission.

**Review Comments to the Author**

**Reviewer #1**: The manuscript attempts to analyze the biofilm-forming capacity of Staphylococcus epidermidis ATCC 35984 on four different platelet concentrate (PC) storage bag materials. While the topic is relevant to the role of biofilms in transfusion safety, the study lacks the data quality, depth, and clarity necessary for publication in PLOS ONE.

**1. Insufficient Data Quality and Depth:**

- The study does not present growth kinetics of S. epidermidis under the tested conditions and only reports biofilm formation. Without corresponding planktonic growth data, it is difficult to interpret the biofilm results.

Response to the reviewer #1: We thank the reviewer for this observation. We would like to clarify that bacterial viability under the tested conditions was assessed in both models but using different approaches. In the PC bag model, only planktonic growth was measured (Figure 3), as sampling for biofilm would have disrupted the system. In contrast, in the coupon model, the viability results shown in Figure 2 represent the total bacterial population, including both planktonic and biofilm-associated cells, recovered together.

Importantly, the purpose of these viability measurements was not only to monitor bacterial proliferation, but also to confirm that the absence of detectable biofilm was not due to a lack of viable bacteria in the system. This distinction is now explicitly stated in the revised manuscript.

We have also clarified the use of the term viability in the Methods and Results sections to ensure it accurately reflects the context—whether referring to planktonic cells alone or to the total bacterial population (planktonic + biofilm).

**Modification of section “Quantification of bacterial viability by colony count” of the materials and methods (Line 217):** *For PC bags, an aliquot of the growth medium was collected on each day of analysis to quantify planktonic bacterial viability only, as biofilm sampling have disrupted the system. For coupons, the entire growth medium in each well was transferred into a tube along with its corresponding plastic coupon. ~~For both growth conditions,~~ T~~t~~he tube was ~~mixed by~~ vortexed for ten seconds to dislodge both planktonic and surface-adhered bacteria, allowing quantification of the total bacterial population.*

*The ~~medium~~ resulted suspensions were~~as~~ serially ~~then~~ diluted and ~~spread~~ plated on plate count agar. The inoculated agar plates were then incubated at 37 ± 1 °C for 16 to 24 hours, and the number of colonies was visually counted. All bacterial concentrations are presented as mean log CFU/ml (n = 3).*

**Modification of the legend for figures 2 and 3 (Line 261 and Line 304):**

***Fig 2. Biofilm formation and viability of S. epidermidis ATCC 35984 on plastic bag coupons.*** *Mean OD by CV assay (A, C, E) and mean total ~~log~~ bacterial concentration (log CFU/ml) (B, D, F) are shown for day 2, 5 and 7 ~~after~~ of incubation in TSB (A, B), PCs (C, D) and plasma (E, F) (n = 3). The shaded gray area represents the threshold for biofilm formation. Viability values represent the total bacterial population recovered from both the medium and the coupon surface (planktonic + biofilm-associated cells).*

***Fig 3. Biofilm formation and viability of S. epidermidis ATCC 35984 in PC storage bags.*** *Mean OD by CV assay (A, C) and mean planktonic ~~log~~ bacterial concentration (log CFU/ml) (B, D) are shown for day 2, 5 and 7 ~~after~~ of incubation in TSB (A, B) and PCs (C, D) (n = 3). Viability values represent planktonic cells only, as biofilm sampling was not performed in bags to preserve system integrity.*

**2. Clarity of Writing:**

- The manuscript's writing is often unclear, making it challenging to follow the methodology and results. Major revisions are required for clarity and flow.

Response to the reviewer #1: We appreciate the reviewer’s feedback regarding the clarity of the manuscript. In response, we have made substantial revisions throughout the text to improve clarity and flow. Specifically:

- We clarified the details of the initial inoculum in the Materials and Methods section.
- We emphasized the distinction of planktonic and total viability.
- We highlighted the contribution of cumulative biofilm analyses (AUC) to better capture biofilm development over time.
- We reinforced the statistical analysis to support our conclusions more robustly.

We believe these changes significantly enhance the readability and overall coherence of the manuscript.

**3. Experimental Design Limitations:**

- Biofilm formation is expected to differ between TSB, plasma, and PC.

It's unclear how long S. epidermidis was cultured in TSB before inoculation into plasma or PCs, or whether inoculation was performed from TSB culture or glycerol stock. This information is critical.

Response to the reviewer #1**:** As described in the Materials and Methods section, the inoculum was prepared from fresh *S. epidermidis* cultures grown in TSB and adjusted based on optical density (OD₆₀₀) measurements. Cultures were harvested during the exponential growth phase to ensure physiological consistency, which is particularly relevant for biofilm formation. Inoculation into plasma or platelet concentrates (PCs) was performed using these fresh cultures, not from glycerol stocks. This approach aligns with the ISO 4768:2023(E) standard. We have revised the text to make this process more explicit and avoid any ambiguity.

**Change made Line 149**: *An agar plate was inoculated from a frozen culture of S. epidermidis ATCC 35984 and incubated at 37°C ± 1 for 24 hours. A fresh culture ~~of S. epidermidis ATCC 35984~~ was then obtained by inoculating 10 ml of TSB with a few colonies from the agar plate.*

**Explanatory sentence added Line 160**: *Fresh cultures were used instead of frozen stocks to ensure physiological consistency, particularly maintaining bacteria in the exponential growth phase, which is known to be critical for initiating robust and reproducible biofilm formation.*

- Ethical Committee Clarification (Line 101):

Please specify the number. “Héma-Québec's ethics committee”

**Add Line 108-109**: This study was approved by Héma-Québec's ethics committee (approval number: CER-2022-013).

**4. Misplaced or Ambiguous Text:**

- Line 122: Reference 25 is included as a full title in the main text. This should be rephrased or removed.

**The title of the standard has been removed (Line 129)** : *Strain ATCC 35984 was selected to comply with the ISO 4768:2023(E) standardization ~~"Measurement method of anti-biofilm activity on plastic and other non-porous surfaces"~~ [25] and because it has been widely used in previous biofilm related studies [6,26,27].*

- Abstract: It states that four materials were tested, but only two (PVC and EVA) are listed. Please clarify.

**Addition of material details to the abstract (Line 40):** *~~The~~* *Four surface material conditions were tested: ~~included~~ polyvinyl chloride (PVC) plasticized with butyryl trihexyl citrate (BTHC) – both smooth and rough sides, PVC plasticized with tri-(2-ethylhexyl)-trimellitate (TEHTM), and ethylene vinyl acetate (EVA) bags. Both coupons and bags made from each material were used in the experiments.*

- Line 139: "At a maximum" it should specify "OD600" for bacterial growth.

**Change made Line 154**: *" and the bacterial concentration was estimated by measuring the optical density at 600 nm using a spectrophotometer (Thermo Scientific, Genesys 10S UV-Vis)."*

- Line 152: Clarify whether bacteria were inoculated into plasma or platelets, and from what source (TSB, glycerol stock).

**Change made Line 149**: *An agar plate was inoculated from a frozen culture of S. epidermidis ATCC 35984 and incubated at 37°C ± 1 for 24 hours. A fresh culture ~~of S. epidermidis ATCC 35984~~ was then obtained by inoculating 10 ml of TSB with a few colonies from the agar plate.*

- Line 159: Remove double brackets: “([20–24 °C])”.

**Change made Line 185**: (20–24 °C).

**5. Figures and Data Presentation:**

- Figure 1B: The image is not clear and needs improvement.

Response to reviewer #1: We acknowledge that the image may appear less detailed; however, this is inherent to the nature of the material being imaged. The plastic surface is smooth and largely featureless, with only minor imperfections, which naturally results in a lower contrast and fewer visible structures at this magnification.

We have verified that the image is in focus and of appropriate quality. It is important to maintain the same magnification across all images in the figure to ensure consistency and allow for accurate comparison between samples. Altering the magnification or applying excessive image processing could misrepresent the material’s actual appearance.

- Figure 2E: The X-axis labeling is inadequate; values are not fully visible.

Response to reviewer #1: Thank you for your observation. We believe there may have been a misunderstanding, as the issue appears to concern the Y-axis rather than the X-axis. The Y-axis represents biofilm quantity measured by optical density, and the values are indeed very low across all conditions.

We intentionally maintained the Y-axis scale between 0 and 3 to ensure consistency with the other graphs in the figure. This uniform scale allows for direct visual comparison and clearly illustrates that biofilm formation is negligible in this condition, remaining well below the detection limit threshold. Additionally, no statistically significant differences were observed between the tested conditions, which further supports the appropriateness of the current scale.

- Initial Log (CFU/mL) is different in TSB and PCs.

Response to reviewer #1: We acknowledge that slight variations in the initial bacterial concentration may occur. As described in the Materials and Methods section, the inoculum was prepared based on optical density measurements at 600 nm, which is a widely used and practical method for estimating bacterial concentration in real time. This approach allowed us to prepare fresh cultures in the exponential growth phase and adjust the inoculum to the target concentration recommended by the ISO 4768:2023(E) standard (~10⁴ CFU/ml). We chose this method over the use of frozen bacterial stocks for several reasons, but the principal is the fresh cultures ensure physiological consistency, particularly in the exponential phase, which is relevant for biofilm formation.

**Explanatory sentence added (Line 160)**: *Fresh cultures were used instead of frozen stocks to ensure physiological consistency, particularly maintaining bacteria in the exponential growth phase, which is known to be critical for initiating robust and reproducible biofilm formation.*

- Figure 4: It is unclear how presenting biofilm formation as area under the curve (AUC) adds new insight. This seems repetitive of prior data.

Response to reviewer #1: Thank you for this observation. We agree that the day-by-day data already provide detailed insight into biofilm formation dynamics. However, we included the AUC analysis to offer a complementary, integrative perspective on the overall biofilm formation behavior across the full 7-day incubation period. This approach helps to mitigate the influence of day-to-day variability or outlier values, highlight cumulative trends that may not reach statistical significance at individual time points but become evident when considered over time and facilitate direct comparisons between plastics by summarizing the total biofilm burden. To clarify this added value, we have revised the corresponding paragraph in the Results section to better explain the rationale for using AUC and how it complements the point-by-point analysis.

**Changes made Line 313**: *To ~~conduct~~ further explore ~~a more in-depth analysis of~~ the ~~same data~~ cumulative biofilm formation behavior over the entire incubation period, we ~~assessed~~ calculated ~~cumulative biofilm growth on the surface of plastic coupons over the seven-day storage period by calculating~~ the AUC for each condition ~~observed profiles~~ (****Fig 4****). While individual time-point comparisons provide insight into the kinetics of biofilm development, AUC offers a global measure of biofilm accumulation, reducing the impact of variability at specific time points. ~~This approach helps mitigate the influence of outlier values.~~ This integrative approach revealed that EVA ~~was~~ had a significantly higher cumulative biofilm formation in TSB compared to S-PVC-BTHC and PVC-TEHTM (p < 0.01) (****Fig 4A****), and that R-PVC-BTHC accumulated more biofilm than ~~that for the other plastics, but it was statistically significant only when compared with~~ S-PVC-BTHC in PCs (p < 0.05) (****Fig 4B****). When comparing the AUC between TSB and PCs, a trend emerges indicating that biofilm levels are generally higher in PCs than in TSB, except for EVA. These findings support the trends observed in the time-point data and reinforce the influence of surface texture and plastic type on biofilm development.*

**Change to Figure 4 legend (Line 330):** *Fig 4. Cumulative biofilm formation of S. epidermidis ATCC 35984 on plastic bag coupons, expressed as AUC. AUC values were calculated ~~are obtained~~ from CV assay absorbance profiles ~~curves~~ over the seven-day ~~storage~~ incubation period in ~~for~~ TSB (A) and PCs (B). Statistically significant differences were observed between EVA and S-PVC-BTHC and PVC-TEHTM in TSB (**p < 0.01) and between R-PVC-BTHC and S-PVC-BTHC in PCs (*p < 0.05).*

- Authors should provide images of the PC coupons and storage bags used.

Response to reviewer #1: As requested, we have added images of the platelet concentrate (PC) storage bags and coupons used in the study to the Supplementary Information (Fig_S1). These images include the physical dimensions, internal surface areas, and volume-to-surface area ratios for each bag format. This addition aims to provide a clearer understanding of the experimental setup, and the characteristics of the storage containers used.

Here is the legend of the new figure added to the pockets and coupons used in the study:

***S1 Fig. Schematic representation of the platelet concentrate storage bags used in this study.*** *Two formats are shown: full-size bags, used for experiments involving TSB and plasma, and reduced-volume bags, created by heat-sealing full-size bags, used for experiments with PCs. This figure illustrates the physical dimensions of the BTHC (A, D), CLX (B, E) and EVA (C, F) bags (cm), the internal surface areas available for gas exchange (cm² for surface areas), and the total volume (ml) after the addition of the tested storage medium.*

**Additional Clarifications Required:**

• Statistical analysis: Ensure p-values or effect sizes are reported for key comparisons.

Response to reviewer #1: We thank the reviewer for this excellent suggestion. Given our relatively small sample size, we agree that reporting effect sizes in addition to p-values provides valuable insight into the magnitude of observed differences. We have therefore calculated and included Cohen’s d values for key pairwise comparisons throughout the Results section. These effect sizes help to contextualize the biological relevance of our findings, even in cases where statistical significance was not reached. The manuscript has been updated accordingly.

**Size effect analysis added Line 254:** *On day 7, although this difference appears similar~~; however~~, no statistically significant differences was~~ere~~ detected (p = 0.14), mainly due to the large standard deviation. However, a large effect size was observed (d = 1.20) between EVA and S-PVC-BTHC, suggesting a potentially meaningful difference in biofilm formation that may warrant further investigation. Overall~~Therefore~~, biofilm formation behavior remained consistent between plastic samples on day 7.*

**Size effect analysis added Line 322**: *This integrative approach revealed that EVA had a significantly higher cumulative biofilm formation in TSB compared to S-PVC-BTHC and PVC-TEHTM (p < 0.01; d = 3.68) (Fig 4A), and that R-PVC-BTHC accumulated more biofilm than S-PVC-BTHC in PCs (p < 0.05; d = 1.96) (Fig 4B).*

• Line 132: Authors mention 2.5 × 2.5 cm coupons. What was the size of the full PC storage bags used?

Response to reviewer #1: As mentioned in our previous response, we have added images of the platelet concentrate (PC) storage bags and coupons to the Supplementary Information (S1_Fig.).

• Line 140: Confirm whether the culture was adjusted to 10⁴ CFU/ml in all conditions (TSB, PC, plasma).

Response to reviewer #1: While OD600 provides a reliable estimate, slight variations in the initial bacterial load may occur due to the inherent limitations of this method (3,5 to 5,5 log). We have clarified this point in the revised manuscript (Line 149). It can also be observed that growth reaches a plateau at similar levels regardless of the initial inoculum. Therefore, we do not believe that differences in initial concentration have an impact on biofilm formation.

**Change made Line 155:** *The bacterial culture was ~~then~~ adjusted to an estimated concentration of 10⁴ CFU/ml, as recommended by the ISO 4768:2023(E) standard, in all experimental conditions (TSB, plasma, or PCs)*. […] *Variations in the initial bacterial load may have occurred due to the inherent variability of spectrophotometric estimation.*

- Language Corrections:

For example, "Significative" → "significant"

**Language correction made:** The change was made twice for "Significative" → "significant"

**Reviewer #2: Reviewer Comments**

• The work is interesting however there are few suggestions for the improvement of the manuscript.

• The abstract says that - The four materials tested included plasticized polyvinyl chloride (PVC) and ethylene vinyl acetate (EVA) bags. To the reader it would appear that only two materials are mentioned here. Though the materials are only two PVC and EVA, however you have tested with PVC- BTHC-Smooth, PVC- BTHC-Rough, PVC-TEHTM and EVA. So, it would be better to clearly mention this in the abstract.

**• It is important to mention the following in the abstract otherwise it would be confusing for the reader:**

o Coupons and bags of each of the materials have been used.

o The PVC-BTHC coupons have both a smooth and a rough side.

Response to reviewer #2:

**Addition of material details to the abstract (Line 40):** *~~The~~* *Four surface material conditions were tested: ~~included~~ polyvinyl chloride (PVC) plasticized with butyryl trihexyl citrate (BTHC) – both smooth and rough sides, PVC plasticized with tri-(2-ethylhexyl)-trimellitate (TEHTM), and ethylene vinyl acetate (EVA) bags. Both coupons and bags made from each material were used in the experiments.*

**• Abstract: … rough side of PVC-BTHC showed significative biofilm formation … / Please correct as … rough side of PVC-BTHC showed significant biofilm formation.**

**Corrected:** (Line 48) significative → significant

**• Methods: Preparation of the plastic coupons could be given as a separate section or included in the PC bags section as PC bags and coupons.**

Response to reviewer #2: We agree that separating the preparation of plastic coupons into its own subsection would improve the clarity of the Materials and Methods section. We have revised the manuscript accordingly to better distinguish the preparation steps for both the coupons and the full-size PC bags.

**Section added line 142**: ***Preparation of plastic coupons***

*The PC storage bags were cut into square coupons of 2.5 cm × 2.5 cm. […]*

**• Results and Discussion: Though the authors have explained in the manuscript why there is no biofilm formation when Staphylococcus epidermidis is grown in plasma with coupons literature does reveal that plasma is a key regulator of S. epidermidis biofilm formation. Therefore, this aspect should be justified clearly.**

Response to reviewer #2: We thank the reviewer for this insightful comment. While it is well established in the literature that plasma components can promote *S. epidermidis* biofilm formation, our results suggest that this effect may be context dependent. In our study, biofilm formation was assessed on plastic materials in the presence of human plasma under specific conditions: time, temperature, etc. It is possible that the interaction between plasma proteins and the plastic surface, or the dynamic conditions of our model, limited biofilm development. Additionally, the strain used in our study may respond differently to plasma exposure compared to those used in previous reports. We have revised the Discussion section to better highlight these possible explanations and to acknowledge the apparent discrepancy with existing literature.

**Changes made Line 365**: *While several studies have reported that plasma can promote S. epidermidis biofilm formation by providing adhesion-facilitating proteins such as fibrinogen and fibronectin, our findings show a complete absence of biofilm in plasma under the tested conditions. This discrepancy may be explained by the dual nature of plasma components. In addition to adhesion-promoting proteins, ~~Conversely,~~ plasma contains antimicrobial peptides and neutralizing antibodies ~~[27]~~ that can inhibit bacterial growth and limit biofilm formation [27]. These positively charged peptides and proteins interact with negatively charged bacterial membranes, increasing their permeability and leading to bacterial death [33], while ~~Neutralizing~~ antibodies may block key ~~can bind to receptors and proteins involved in~~ biofilm-associated proteins ~~formation~~ such as the accumulation-associated protein [34]. In our study, bacterial viability in plasma was significantly reduced, suggesting that these inhibitory effects predominated. These factors likely explain the reduced bacterial growth and absence of biofilm formation in plasma. ~~Surprisingly~~ In contrast, ~~this effect is not observed in~~ PCs, which are suspended in 100% plasma, ~~this~~ consistently supported biofilm formation. This suggests that platelets may counteract the antimicrobial effects of plasma ~~play a crucial role~~ by ~~in~~ promoting biofilm bacterial adhesion and protecting bacteria against from ~~plasma-derived antimicrobial properties~~ host defenses [28, 29].*
