# Supplementary material for: Monitoring of Staphylococcus epidermidis biofilm formation on platelet storage bag surfaces
Source: PLoS One. 2025 Oct 22;20(10):e0333558. doi: 10.1371/journal.pone.0333558 (PMC12543116; doi:10.1371/journal.pone.0333558)
Supplement: Attachment — Submitted filename: Response_to_Reviewers_auresp_2.docx [file pone.0333558.s006.docx]

We sincerely thank the reviewers for the thoughtful and constructive feedback. Your comments and suggestions have helped us clarify key aspects of our study and improve the overall quality of the manuscript. We have addressed each of your concerns in detail below. In addition to the specific revisions, we have made multiple changes to the manuscript to improve its flow and make it more concise due to the text added.

Note: The reviewer comments are listed in blue below.

**Review Comments to the Author**

SPECIFIC COMMENTS

ABSTRACT

**Reviewer #3 – Comment #1**

Line 28. PCs are stored at "20-24 C" is preferably than "room temperature" as ambient temperature can change in different locations.

Change made Line 28: *Platelet concentrates (PCs) are stored at ~~room temperature~~ 20-24˚C, favoring bacterial growth and making those products susceptible to contamination.*

**Reviewer #3 – Comment #2**

Line 29. The statement “making those products susceptible to contamination” is inaccurate as PC storage at room temperature does not make the product more susceptible to contamination, it potentially favors growth of bacteria already introduced during venipuncture.

Change made Line 29: *Platelet concentrates (PCs) are stored at 20-24˚C in a biologically favorable environment that may support bacterial growth.*

**Reviewer #3 – Comment #3**

Line 48. From the results presented (Line 43: “similar biofilm amount across PC bag materials in the presence of platelets”), it is not clear how the following conclusion can be made “However, platelet bag materials limit biofilm formation under standard blood bank storage conditions, reducing the risk of undetected bacterial contamination”. Please revise.

Response to the reviewer: We agree that the original phrasing may have been misleading. Our intention was to highlight that the environment within platelet storage bags, characterized by continuous agitation and reduced oxygen levels, limits biofilm formation, even when using materials that promote biofilm under static conditions. We have revised the sentence to clarify this point:

Change made Line 49: *However, during blood bank storage (i.e., continuous agitation and reduced oxygen levels), biofilm formation is limited, regardless of the platelet bag material, thereby reducing the risk of undetected bacterial contamination.*

INTRODUCTION

**Reviewer #3 – Comment #1**

Line 52. Please see comment above about “room temperature”

Change made Line 51**:** *The storage conditions for platelet concentrates (PCs) in blood services ~~room temperature~~*—*20-24˚C under constant agitation*—*create an environment conducive to bacterial proliferation.*

**Reviewer #3 – Comment #2**

Line 53. “Consequently, PCs are more at risk of contamination” is incorrect. Please clarify to state that PCs are more CONDUCIVE to proliferation of bacteria introduced during blood donation due to their storage conditions.

Change made Line 52: *The storage conditions for platelet concentrates (PCs) in blood services, 20-24˚C under constant agitation, create an environment conducive to bacterial proliferation. Consequently, blood services systematically test all PCs prior to their clinical use [1–3].*

**Reviewer #3 – Comment #3**

Line 62. References 11-14 support the incidence of TTBIs post-implementation of culture systems not pathogen reduction. The safety risk of PCs screened with culture methods or treated with pathogen reduction technologies is not the same and should not be combined as one category.

Response to the reviewer: We have revised the relevant paragraph in the introduction to clearly distinguish between these two strategies. Specifically, we now state that TTBI cases referenced in [11–14] occurred despite the implementation of culture-based screening systems, and do not reflect the residual risk associated with PRTs. Furthermore, we have added new references [15–17] to support the discussion of PRTs and their specific limitations, including the influence of bacterial load, timing of application, and resistance mechanisms such as biofilm formation.

Added the following text Line 61: *While both strategies have significantly improved transfusion safety, they address bacterial contamination through distinct mechanisms and face different limitations. Culture-based methods may fail to detect slow-growing or biofilm-forming bacteria such as S. epidermidis, leading to false-negative results [11–14]. In contrast, PRTs aim to inactivate a broad range of pathogens, including bacteria, viruses, and parasites. Their efficacy can be influenced by factors such as the initial bacterial load, the timing of application, and the resistance of certain species, including those capable of forming biofilms [15]. Moreover, rare cases of transfusion-transmitted bacterial infections (TTBIs) have also been reported following PRT implementation, often due to post-treatment contamination or limitations in the inactivation process [16, 17].*

MATERIALS AND METHODS

**Reviewer #3 – Comment #1**

How many biological repetitions of the experiments were done?

Response to the reviewer: All experiments were performed in triplicate and repeated independently three times (n=3). This means that each condition was tested in three independent biological replicates, each including technical triplicates, to ensure the robustness and reproducibility of the results.

Change made Line 139: *For each condition, experiments were conducted in technical triplicates and repeated independently three times (i.e., three biological replicates; n = 3).*

Change made at the end of each Figure legend: *Error bars represent the standard deviation across three biological replicates.*

**Reviewer #3 – Comment #2**

Line 126. Why was TSB not supplemented with 0.5-1.0% of glucose? It is well known that glucose supplementation of TSB increases biofilm formation by staphylococci.

Response to the reviewer: We thank the reviewer for this relevant observation. While glucose supplementation is indeed known to enhance biofilm formation in *Staphylococcus* species, we chose to use unmodified TSB (containing 0.25% glucose) to align with the ISO 4768:2023(E) standard. Moreover, *S. epidermidis* ATCC 35984 is known to form robust biofilms even in standard TSB, which was confirmed in our experiments. In addition, we conducted experiments using TSB supplemented with 0.5% and 1.0% glucose to evaluate its impact on biofilm formation with this strain. Surprisingly, we did not observe a significant increase in biofilm biomass compared to standard TSB. These findings are consistent with those reported by Fonseca et al. (<https://doi.org/10.1016/j.microb.2025.100401>), using *S. epidermidis* ATCC 35984, who demonstrated that glucose supplementation does not universally enhance biofilm formation and that the response may vary depending on strain-specific factors and environmental conditions. We have specified this information in the text.

Added the following Line 135: *Under the experimental conditions used in this study, S.  epidermidis ATCC 35984 consistently formed biofilms in TSB without additional glucose supplementation.*

**Reviewer #3 – Comment #3**

Line 130. Which type of plates were used to test PC bags’ coupons? How was bacterial attachment directly to the plates avoided? Was interference of attachment to the plate with attachment to the coupons considered?

Response to the reviewer: The plastic coupons were placed in standard sterile six-well polystyrene plates for incubation and staining procedures. To minimize bacterial attachment to the plate surface, the coupons were carefully transferred to new sterile plates after washing steps, and only the biofilm formed on the coupons themselves was analyzed. In addition, blank controls (medium without bacterial inoculation) were included to account for any non-specific adhesion to the plate or plastic surfaces. The possibility of interference from bacterial attachment to the plate was considered and mitigated by focusing exclusively on the biofilm eluted from the coupons during the crystal violet assay.

Change made Line 145: *Coupons were affixed to the bottom of sterile six-well polystyrene plates using Steri-Strips (Cardinal Health, cat. 3MR1540).*

Change made Line 185: *After incubation, the coupons were immersed in sterile water and washed by gently moving them back and forth about five times before being transferred to a new sterile plate to minimize potential bacterial attachment to the original plate surface. Only the biofilm formed on the coupons was analyzed, and blank controls were included to account for non-specific adhesion.*

**Reviewer #3 – Comment #4**

Line 131. Were PCs stored in a platelet incubator? If not, please provide storage conditions (temperature and agitation).

Change made Line 176: *The bags were incubated at ~~room temperature~~ 20-24 °C on a platelet agitator (70 ± 1 cycles per minute with a 3.8 cm amplitude) for seven days.*

**Reviewer #3 – Comment #5**

Line 145. How was estimation of bacterial load adjusted without colony counts?

Response to the reviewer: The bacterial concentration was initially estimated by spectrophotometry at 600 nm using a Genesys 10S UV-vis spectrophotometer. The culture was adjusted to an estimated 10⁴ CFU/ml in accordance with the ISO 4768:2023(E) standard. To validate this estimation, we performed colony counts on the adjusted suspension to confirm the actual bacterial load prior to inoculation. This approach ensured both consistency and accuracy of the inoculum across all experimental conditions.

Change made line 155: *The bacterial concentration was estimated by spectrophotometry (Thermo Scientific, Genesys 10S UV-Vis) at 600 nm and adjusted to an estimated 104 colony-forming units (CFU)/ml, in accordance with the ISO 4768:2023(E) standard. To validate this estimation, colony counts were performed on the adjusted suspension in all experimental conditions (TSB, plasma, or PCs).*

**Reviewer #3 – Comment #6**

Line 149. Please elaborate in your statement “Variations in the initial bacterial load may have occurred due to the inherent variability of spectrophotometric estimation” Was the variability estimated? It is known that working with a robust biofilm former strain such as ATCC 35984 is challenging as bacterial aggregates are difficult to resuspend resulting in great variability of bacterial counts. Why were not biofilm positive and negative PC isolates of S. epidermidis used instead of commercial strains?

Response to the reviewer: The statement regarding variability in bacterial load refers to the known limitations of spectrophotometric estimation, particularly when working with biofilm-forming strains such as *S. epidermidis* ATCC 35984, which tend to form aggregates that can affect optical density readings. To address this, we performed colony counts on the adjusted suspensions to validate the estimated bacterial concentration prior to inoculation, as now clarified in the revised Materials and Methods section.

Regarding the choice of strains, *S. epidermidis* ATCC 35984 was selected in accordance with the ISO 4768:2023(E) standard, which recommends this strain for evaluating biofilm formation on plastic and other non-porous surfaces. This strain is widely recognized and used in biofilm-related studies due to its strong and reproducible biofilm-forming capacity mainly onto polymeric materials. Our objective was not to assess the biofilm-forming potential of different clinical isolates, but rather to compare biofilm formation across different plastic materials used in PC storage bags. Using a well-characterized, strong biofilm-forming strain ensured consistent biofilm development and allowed us to focus on the influence of the material composition and surface texture. A more detailed rationale for strain selection is provided in the Discussion section comments (Discussion - comment #6).

Added the following text Line 161: *Variations in the initial bacterial load (±1 log) may have occurred due to the inherent limitations of spectrophotometric estimation, particularly when working with S. epidermidis ATCC 35984. This strong biofilm-forming strain tends to aggregate, complicating resuspension and thereby affecting the accuracy of optical density measurements.*

**Reviewer #3 – Comment #7**

Line 157. Why were the coupons incubated at 22+/- 1C? Why were they incubated with no agitation?

Response to the reviewer: The coupons were incubated at 22 °C, which corresponds to the setpoint of our temperature-controlled incubator for platelet storage. This temperature was specifically chosen to fall within the acceptable storage range for PCs, which is 20–24 °C, as recommended by blood bank standards. To ensure clarity and consistency throughout the manuscript, we have revised the text to uniformly refer to this condition as “room temperature (20–24 °C)”.

The decision to incubate without agitation was based on the protocol outlined in the ISO 4768:2023(E) standard, which recommends static conditions for biofilm formation assays. This approach was chosen to replicate the passive environment in which biofilms may develop on plastic surfaces during storage.

Additionally, we conducted preliminary experiments under agitated conditions (see Supporting Information, Fig S1), and no significant biofilm formation was observed. These results further supported our decision to proceed with static incubation, as agitation appeared to inhibit biofilm development in our model.

We hope this clarification addresses the reviewer’s concerns and reinforces the rationale behind our methodological choices.

Change made Line 168: *The plates were then incubated at ~~22±1~~ 20-24 °C with ≥ 90 % relative humidity for seven days without agitation.*

**Reviewer #3 – Comment #8**

Line 171. How many times the coupons were washed?

Change made Line 185: *After incubation, the coupons were ~~washed in sterile water~~* *immersed in sterile water and washed by gently moving them back and forth about five times before being ~~and~~ transferred to a new sterile plate.*

**Reviewer #3 – Comment #9**

Line 189. Biofilm-negative staphylococcal strains convert to a biofilm positive phenotype when grown in PCs, why was the ATCC 12228 strain then used as a negative control for biofilm formation assays in platelet products?

Response to the reviewer: We appreciate the reviewer’s comment and understand the concern regarding the use of *S. epidermidis* ATCC 12228 as a negative control. As noted in comment #3 of the results section, this strain was used in TSB, where it is well characterized as biofilm-negative under standard laboratory conditions. This choice was made to establish a consistent and conservative threshold (ODc) for biofilm detection across different plastics and media, in accordance with ISO 4768:2023(E).

While some studies (<https://doi.org/10.1111/j.1537-2995.2007.01249.x>; <https://doi.org/10.1111/trf.12308>) have reported biofilm formation by ATCC 12228 in PCs, we did not test this strain in PCs in the present study. Our goal was to define a reproducible baseline, and non-spiked PCs proved too low and variable for this purpose (see comment #3 – section Results). Therefore, ATCC 12228 in TSB was selected as a reliable reference condition.

RESULTS

**Reviewer #3 – Comment #1**

Lines 241 and 282. Please replace “concentration” with “load”

Change made Line 254: *Bacterial viability was comparable across all plastics throughout the storage period, with a final ~~concentration~~ load of ~8.5 log CFU/ml (****Fig 2B****).*

Change made Line 296: *Despite the small amount of biofilm, bacterial growth remained consistent, bacterial ~~concentration~~ load being ~7.0 log CFU/ml across all materials on day 7 (****Fig 3D****).*

**Reviewer #3 – Comment #2**

Line 293. Regarding the statement “Biofilm formation on coupons between TSB and PCs was compared for each type of plastic and each day of analysis; however, no statistical differences were observed”, please provide the p values for each day between TSB and PCs.

Change made Line 308: *Biofilm formation on coupons between TSB and PCs was compared for each type of plastic and each day of analysis; however, no statistical differences were observed (p > 0.05).*

**Reviewer #3 – Comment #3**

Figure 2 shows differences between media and PCs. Even if there are no statistical differences, biologically, what is the meaning of having values within the threshold zone for some samples and not others? It is concerning that biofilm formation results with the ATCC 12228 strain were used to establish a threshold when this strain is known to form biofilms in PCs and therefore can tamper the interpretation of the results. Non-spiked PCs can be used as baseline for biofilm formation assays in this milieu.

Response to the reviewer: We thank the reviewer for this insightful comment. The threshold (ODc) was established using the *S. epidermidis* ATCC 12228 strain in TSB, where this strain is well characterized as biofilm-negative under standard laboratory conditions. This approach was adopted following a previous reviewer’s suggestion to provide a consistent and conservative baseline for detecting biofilm formation across different plastics and media (>ODc = positive results).

We acknowledge that some studies have reported that ATCC 12228 may form biofilms in PCs under certain conditions (<https://doi.org/10.1111/j.1537-2995.2007.01249.x>; <https://doi.org/10.1111/trf.12308>). However, we did not test this strain in PCs in the present study. That said, we initially considered using non-spiked PCs as a baseline, but the resulting absorbance values were extremely low and highly variable, making it difficult to define a reliable threshold. For this reason, we opted to use the ATCC 12228 strain in TSB, where its behavior is well established and reproducible.

Regarding values near or within the threshold zone, we agree that these may reflect early or weak biofilm formation or variability in adhesion depending on the plastic or medium. While not statistically significant, these trends were further explored using AUC analysis, which integrates biofilm formation over time and revealed meaningful differences between plastics and media.

**Reviewer #3 – Comment #4**

Please define asterisks in figure legends.

Response to the reviewer: Asterisks were already defined in figure legends 2 and 4 *(**p < 0.01)* and *(*p < 0.05)*, but no statistically significant differences were observed in figure 3. A sentence has been added to make sure this was clear.

Change made Figure 3 legend: *Biofilm was assayed on R-PVC-BTHC (**), S-PVC-BTHC (**), PVC-TEHTM (**) and EVA (**) are represented. No statistically significant differences were observed (p ˃ 0.05).*

Discussion

**Reviewer #3 – Comment #1**

Line 324. Please explain your statement “No biofilm was observed in plasma, which may be partly due to plasma's antimicrobial peptides and neutralizing antibodies that limit bacterial growth and, consequently, S. epidermidis biofilm formation”. PCs used in the study were suspended in 100% plasma and therefore they also had antimicrobial peptides and antibodies. Please consider discussing the absence of platelets in plasma, which could be the limiting factor for biofilm formation.

Response to the reviewer: We thank the reviewer for this insightful comment. Initially, the first paragraph of the discussion aimed to summarize the main findings, including the absence of biofilm in plasma and its presence in PCs. However, to avoid redundancy and improve clarity, we have removed this paragraph. The role of antimicrobial peptides and antibodies in plasma is discussed in more detail later in the discussion (Line 361), where we highlight the potential of platelets to counteract these antimicrobial effects and promote biofilm formation.

Added the following text Line 366*: Another important factor to consider is the absence of platelets in plasma, which could limit bacterial adhesion and biofilm development.*

**Reviewer #3 – Comment #2**

Line 326. Please explain what the difference in gas accessibility between plates and PC bags is. PC containers are made of gas permeable material and are stored under agitation. One would expect that there is more oxygen accessibility in the bags compared to the coupons stored in plates made of hard plastic.

Response to the reviewer: We appreciate the reviewer’s comment and the opportunity to clarify this point. While PC containers are indeed made of gas-permeable materials and stored under agitation, they are sealed systems, meaning that gas exchange occurs exclusively through the plastic material. In contrast, the plates used in our experiments were covered with loosely fitted lids, similar to Petri dishes, allowing direct gas exchange between the atmosphere and the internal environment through the gap between the lid and the plate.

Although the PC bags are designed to allow oxygen diffusion, the permeability of the plastic is inherently lower than the open-air interface provided by the plate lids. Therefore, despite the agitation in PC storage, the oxygen accessibility in plates is greater due to the open-air exposure. This setup was chosen to ensure that oxygen availability would not be a limiting factor in biofilm formation on the coupons.

We hope this explanation clarifies the rationale behind our experimental design and addresses the reviewer’s concern.

Added the following text Line 376: *Secondly, although PC storage conditions are considered aerobic [43], oxygen availability may differ between PC storage and plates due to differences in gas exchange mechanisms. While PC bags are sealed and rely on gas diffusion through the plastic, the polystyrene plates are loosely covered, allowing passive gas exchange with ambient air and potentially greater oxygen accessibility. Our supplementary experiments (S1 Table) showed that biofilm formation was inhibited under low oxygen conditions, while bacterial growth remained unaffected, indirectly supporting the idea that oxygen availability may influence biofilm development. Although agitation in PC bag storage conditions may enhance mixing, it does not compensate for the limited surface area available for gas diffusion. Future studies should include direct measurements of oxygen concentration in both systems to validate this hypothesis. While S. epidermidis can form biofilms under aerobic conditions by creating anaerobic microenvironments within the biofilm itself [45,46], external oxygen availability may still influence the initiation and extent of biofilm development.*

**Reviewer #3 – Comment #3**

Line 341. For the statement “While several studies have reported that plasma can promote S. epidermidis biofilm formation by providing adhesion facilitating proteins such as fibrinogen and fibronectin, our findings show a complete absence of biofilm in plasma under the tested conditions.” Please provide supporting references as the interpretation of published literature may be inaccurate. While plasma proteins promote bacterial adhesion, mature biofilms are formed in the presence of platelets.

Response to the reviewer: We agree that references should have been added to support this statement. Two references supporting maturation of biofilm of *S. epidermidis* in human plasma were added to the statement (<https://doi.org/10.1186/1756-0500-7-457>; <https://doi.org/10.1093/femspd/ftv125>).

Change made Line 358: *While several studies have reported that plasma can promote S. epidermidis biofilm formation by providing adhesion-facilitating proteins such as fibrinogen and fibronectin [39,40], our findings show a complete absence of biofilm in plasma under the tested conditions.*

**Reviewer #3 – Comment #4**

Lines 345-347. Please see the comment above on “antimicrobial peptides and neutralizing antibodies” which are also contained in PCs.

Response to the reviewer: We agree that antimicrobial peptides and antibodies are present in both plasma and PCs. To address this, we have revised the relevant section of the discussion to clarify that the presence of platelets in PCs may counteract the antimicrobial effects of plasma, thereby enabling biofilm formation. This nuance is now more clearly articulated to avoid any confusion regarding the role of plasma components (see comment #1 Discussion section).

**Reviewer #3 – Comment #5**

Line 362. Reference 36 does not support the statement “oxygen availability may be lower in PC storage bags than in plates, leading to reduced biofilm development”. Furthermore, the statement is speculative, the authors would need to measure oxygen content in a static plastic culture plate and demonstrate that is higher than in a gas permeable PC bag under agitation.

Response to the reviewer: We acknowledge that Reference 36 does not directly support the statement: “oxygen availability may be lower in PC storage bags than in plates, leading to reduced biofilm development.” To address this, we have revised the manuscript to clarify that this statement is speculative (withdrawal of the reference 36) and based on differences in gas exchange mechanisms between sealed PC bags and loosely covered polystyrene plates.

We have also added a note in the discussion emphasizing that future studies should include direct measurements of oxygen concentration in both systems to validate this hypothesis. Additionally, we refer to our supplementary experiments (S1 Table) showing that biofilm formation was inhibited under low oxygen conditions, while bacterial growth remained unaffected. These findings indirectly support the idea that oxygen availability may influence biofilm development.

Added the following text Line 380: *Our supplementary experiments (****S1 Table****) showed that biofilm formation was inhibited under low oxygen conditions, while bacterial growth remained unaffected, indirectly supporting the idea that oxygen availability may influence biofilm development. […] Future studies should include direct measurements of oxygen concentration in both systems to validate this hypothesis.*

**Reviewer #3 – Comment #6**

Line 375. This limitation could be omitted if the experiments of this study are repeated/validated with transfusion relevant isolates that form biofilms but do not form floating aggregates such as the ones produced by strain ATCC35984.

Response to the reviewer: We thank the reviewer for this suggestion. We acknowledge that the tendency of *S. epidermidis* ATCC 35984 to form floating aggregates may limit the applicability of our findings to other transfusion-relevant strains. However, this strain is recommended by the ISO 4768:2023(E) standard for evaluating biofilm formation on plastic materials and is widely used in studies investigating biofilm development and antibacterial properties in blood products. Its well-characterized biofilm-forming capacity makes it a relevant model for assessing the influence of plastic surfaces under standardized conditions. Nonetheless, we agree that future studies using a broader range of clinically relevant isolates, including those that form surface-attached biofilms, would help validate and expand upon our findings. We have added this clarification to the discussion.

Added the following text Line 403: *This limitation is specific to S. epidermidis ATCC 35984, a strain known to form floating aggregates. However, this strain is recommended by the ISO 4768:2023(E) standard for testing biofilm formation on plastic materials and is widely used in studies on biofilms in blood products and antibacterial surface properties [6,19,30–32,36]. Future studies using transfusion-relevant isolates that form surface-attached biofilms could help overcome this limitation and provide a more comprehensive evaluation of biofilm formation in PC storage bags.*

**Reviewer #3 – Comment #7**

Line 387. PCs produced in PAS may have reduced glucose levels, which the authors could calculate, but glucose is still present.

Response to the reviewer: We thank the reviewer for this important clarification. We agree that glucose is still present in PCs stored in PAS due to residual plasma, which typically accounts for approximately 40% of the final volume. However, the PAS solution itself does not contain glucose in its formulation, and its dilution effect leads to a significantly lower glucose concentration compared to PCs stored in 100% plasma. This reduction in glucose availability may contribute to the observed decrease in bacterial growth and biofilm formation, as glucose is an essential nutrient for many bacterial species. We have revised the discussion to reflect this nuance more accurately.

Added the following text Line 416: *Although residual plasma in PAS-stored PCs provides* *sufficient glucose for platelets, the overall concentration remains significantly lower during storage, potentially limiting bacterial proliferation and biofilm development [47]*.

**Reviewer #3 – Comment #8**

There is no discussion about PC treatment with pathogen reduction technologies, which needs to be taken into consideration in the context of biofilm formation.

Response to the reviewer: We agree that pathogen reduction technologies are highly relevant in the context of biofilm formation in PCs. To address this, we have added a paragraph in the Introduction (lines 64-67) to acknowledge the role of pathogen reduction technologies (PRTs) in transfusion safety and their potential limitations regarding biofilm-forming bacteria such as *S. epidermidis*. Additionally, we have included a perspective in the Discussion section (line 410), noting that although our study did not directly evaluate the impact of PRTs, their influence on biofilm formation warrants further investigation. This represents a valuable direction for future research and complements the findings of our current study.

Change made Line 418: *Further studies are needed to investigate the impact of PAS, EVA-based device materials and PRTs on the biofilm-forming behavior of bacterial strains commonly contaminating PCs.*

**Reviewer #3 – Comment #9**

How different is the protocol of ISO 4768:2023(E) standard compared to published literature?

Response to the reviewer: Our study uses the ISO 4768:2023(E) framework, which standardizes key parameters for biofilm quantification on non-porous surfaces, such as storage bags for blood products, including surface size, reagent volumes, and absorbance wavelength. The standard follows the general steps of crystal violet staining, while tightly controlling specific parameters to ensure consistency and reproducibility. One critical aspect is the absorbance wavelength used for quantification, which is fixed at 590 nm in the ISO protocol. Published methods can vary widely in wavelength selection (e.g., 492–595 nm), often compromising reproducibility. A recent publication of George *et al*. (<https://pubmed.ncbi.nlm.nih.gov/39951131/>) highlights this issue by proposing an extinction coefficient-based adjustment method, demonstrating how inconsistencies in wavelength choice can affect inter-study comparability. By applying ISO 4768, our study contributes to improving the robustness and reproducibility of biofilm evaluation on blood product materials.

Added the following text Line 92: *Even though previous studies have investigated biofilm formation on various plastics used in PC storage bags, such as PVC with different plasticizers [19,20], significant protocol differences, particularly in strain preparation and selection, staining conditions and absorbance wavelength measurements [26,27], make it difficult to compare results between studies.*

**Reviewer #3 – Comment #10**

There is a lack of discussion of the results obtained in the study compared to published literature

(e.g., <https://pubmed.ncbi.nlm.nih.gov/28961393/>; <https://pubmed.ncbi.nlm.nih.gov/30354054/>; <https://pubmed.ncbi.nlm.nih.gov/27554133/>) What is the novelty of this study?

Response to the reviewer: We appreciate the reviewer’s suggestion to better position our findings within the context of existing literature. The cited studies by Hadjesfandiari *et al*. (PMID: 28961393) and Mei *et al*. (PMID: 30354054) focus on the development of advanced surface coatings, antifouling and bactericidal, for platelet storage bags. These works demonstrate the potential of engineered polymer surfaces to prevent bacterial adhesion and biofilm formation, using dopamine chemistry and polymer–nanoparticle interactions to create durable, long-term antibiofilm coatings.

Our study differs in scope and approach. Rather than modifying surfaces, we evaluated existing commercial plastic materials used in blood product devices, including various PVC formulations and EVA, under PC storage conditions. Using the ISO 4768:2023(E) standard, we systematically assessed how surface texture, plastic composition, and environmental factors (e.g., oxygen availability, agitation, surface-to-volume ratio) influence *S. epidermidis* biofilm formation. This provides practical insights into the intrinsic biofilm-promoting properties of unmodified plastics, which are currently in use in transfusion settings.

**In summary, the novelty of our study lies in:**

1. Evaluating biofilm formation on **unmodified, clinically used plastic materials** under PC storage conditions.
2. Demonstrating that **plasma alone does not support biofilm formation**, while PCs do, emphasizing the role of platelets.
3. Identifying **physical and environmental factors** that influence biofilm development in blood product storage.
4. Critically assessing the **limitations of the ISO 4768:2023(E) standard** for whole-bag biofilm analysis.

We have revised the discussion to better reflect these distinctions and added references to the relevant literature. For example:

Add reference Line 347 (PMID: 28961393): *This interpretation is supported by previous findings showing that platelet components can promote bacterial aggregation and biofilm development in blood products [32,33,35,36].*

**Reviewer #3 – Comment #11**

There is a lack of discussion of the results of this study for biofilm formation in TSB vs PCs in relation to previous studies which have shown that biofilm formation by S. epidermidis is enhanced in PCs compared to TSB supplemented with glucose. The results presented herein showed the opposite and a potential explanation is needed.

Response to the reviewer: Thank you for this insightful comment. The aim of our study was to compare biofilm formation in plasma versus PCs, using TSB as a standardized positive control. As expected, strong biofilm formation was observed in TSB, particularly with the ATCC 35984 strain. While previous studies (e.g., Greco et al. 2007) reported enhanced biofilm formation in PCs compared to glucose-supplemented TSB, our preliminary tests did not show increased biofilm formation with glucose. Importantly, methodological differences, such as the use of different biofilm quantification techniques (e.g., absence of crystal violet staining), may explain the discrepancies. Additionally, it is possible that donor variability, storage conditions, or specific plasma protein profiles in our PCs influenced biofilm formation differently. We acknowledge this divergence and believe it highlights the need for standardized protocols and further investigation into the biological mechanisms underlying biofilm development in blood components.

**Reviewer #3 – Comment #12**

Please present results of the ATCC12228 strain in all conditions. Previous studies have shown positive biofilm formation by this strain in PCs. If biofilm formation results with this strain were used as a threshold (line 196), this may explain the reduction in biofilm formation on coupons in PCs compared to TSB and this needs to be discussed.

Response to the reviewer: Thank you for this important observation. We agree that presenting the results of the ATCC 12228 strain across all conditions would provide valuable context. However, as clarified in our response to Comment 3 in the Results section, this strain was not tested in PCs in the present study. The threshold used with the ATCC 12228 strain was established in TSB, where this strain is well characterized as biofilm-negative under standard laboratory conditions. This threshold was then applied uniformly across all conditions.

Importantly, our protocol requires subtracting blank values (i.e., background absorbance) from each condition to isolate the biofilm-specific signal. In practice, unspiked PCs tend to yield higher baseline absorbance than unspiked TSB, likely due to the presence of plasma proteins and platelet aggregates adhering to plastic surfaces. These elevated background values in PCs result in greater subtraction, which can potentially lower the final biofilm readings compared to TSB.

This methodological factor may partially explain why biofilm quantities appear higher in TSB than in PCs, even when the same threshold is applied. We have clarified this point in the revised manuscript to avoid confusion.

Added the following text Line 353: *In accordance with the ISO protocol, blank values were subtracted to isolate biofilm-specific signals. In PCs, higher background absorbance, likely due to plasma proteins and platelet aggregates, leads to greater subtraction, which may partly explain the lower biofilm values observed compared to TSB.*

Materials and methods

**Reviewer 4 – Comment #1**

Supplementary Figure S1: The volume and surface area displayed for each pack pair (for example, A and D) are the same. Should this be the case, considering that the pictures D to F denote a reduced volume pack?

Response to reviewer: Thank you very much for this observation, it is indeed an error. The correct volumes and areas have been revised and will be included in Figure S1 in the updated version of the manuscript.

**Reviewer 4 – Comment #2**

Line 204: "The resulted suspensions…" Suggest this is changed to "resulting" or "resultant" suspensions…

Change made Line 222: *The ~~resulted~~ resulting suspensions were serially diluted and plated on plate count agar.*

Results

**Reviewer 4 – Comment #3**

Captions for figures 2, 3 and 4: Please make clear which measure of variance is being used for the error bars.

Change made at the end of each Figure legend: *Error bars represent the standard deviation across three biological replicates.*
